# Psychological Stress and Functional Endometrial Disorders: Update of Mechanism Insights

**DOI:** 10.3389/fendo.2021.690255

**Published:** 2021-08-03

**Authors:** Jin-xiang Wu, Shu Lin, Shuang-bo Kong

**Affiliations:** ^1^Department of Reproductive Medicine, The Second Affiliated Hospital of Fujian Medical University, Quanzhou, China; ^2^Centre of Neurological and Metabolic Research, The Second Affiliated Hospital of Fujian Medical University, Quanzhou, China; ^3^Diabetes and Metabolism Division, Garvan Institute of Medical Research, Sydney, NSW, Australia; ^4^Department of Obstetrics and Gynecology, The First Affiliated Hospital of Xiamen University, Xiamen, China; ^5^Fujian Provincial Key Laboratory of Reproductive Health Research, School of Medicine, Xiamen University, Xiamen, China

**Keywords:** psychological stress, endometrial receptivity, recurrent implantation failure, human endometrium, infertility

## Abstract

The human endometrium plays a vital role in providing the site for embryo implantation and maintaining the normal development and survival of the embryo. Recent studies have shown that stress is a common factor for the development of unexplained reproductive disorders. The nonreceptive endometrium and disturbed early maternal-fetal interaction might lead to infertility including the repeated embryo implantation failure and recurrent spontaneous abortion, or late pregnancy complications, thereby affecting the quality of life as well as the psychological status of the affected individuals. Additionally, psychological stress might also adversely affect female reproductive health. In recent years, several basic and clinical studies have tried to investigate the harm caused by psychological stress to reproductive health, however, the mechanism is still unclear. Here, we review the relationship between psychological stress and endometrial dysfunction, and its consequent effects on female infertility to provide new insights for clinical therapeutic interventions in the future.

## Introduction

Infertility is a disease characterized by the failure to establish a clinical pregnancy after 12 months of regular and unprotected intercourse. Studies have estimated that approximately 12% of married women have difficulty getting pregnant or maintaining a pregnancy (1). Even with the latest whole-genome sequencing technology to select the high quality embryo, the pregnancy rate for euploid embryo transfer has been found to be approximately 45% in the general infertility population (2, 3) and approximately 60% in patients with donor egg embryos (4) undergoing *in vitro* fertilization-embryo transfer (IVF-ET)practices. In recent years, researchers have focused on the role of endometrium in infertility, assuming that endometrial dysfunction might contribute to a large part of pregnancy failure. Thus, reproductive scientists have performed various procedures to analyze the endometrium of patients with recurrent implantation failure (RIF), including hysteroscopy, scratching of the uterine cavity before embryo transfer. Recently, the high-throughput techniques, such as RNA-seq has been utilized to identify new biomarkers of endometrial receptivity to improve the clinical pregnancy rate in IVF-ET technology. However, there have been no relevant advances in diagnosing and improving endometrial receptivity at the molecular level.

The human endometrium is a highly dynamic tissue that is cyclically shed, regenerated, and remodeled. This is mainly regulated by changes in estrogen and progesterone secreted by the ovaries, and the endometrium is only receptive to implantation for a few days during the midpoint of the menstrual cycle (5). Spatiotemporal changes in the endometrium are tightly controlled by cyclic regulation of endocrine hormones from the hypothalamic-pituitary-ovarian axis as well as paracrine morphogens, cytokines, and growth factors generated by the different cellular constituents of the endometrium, including epithelial cells, stromal cells, local immune cells, and the vasculature (6–8). Any factors that might disturb paracrine and autocrine signaling pathways that regulate endometrial function might cause endometrial dysfunction. Embryo implantation and the following decidualization are essential for a successful pregnancy. If the implantation fails, endometrium will manifest as menstruation, followed by regeneration and repair, to prepare for the next implantation window (6). Studies have estimated that approximately one-third of implantation failures occur due to embryos, and the remaining two-thirds are attributed to poor endometrial reception and altered embryo-endometrial dialogue (9–11).

Female infertility has been shown to be associated with high levels of anxiety and depression. Recently, it was reported that mental illness could also increase the incidence of infertility and undesirable pregnancy, such as repeated implantation failure and miscarriage (12–14). Moreover, psychological stress, depression, and anxiety during pregnancy are thought to affect the development of the fetus and might have a long-term impact on the prognosis of the offspring (15). Additionally, numerous studies have shown the efficacy of psychological interventions in reducing psychological distress as well as in increasing pregnancy rates (16, 17). Hormones are known to regulate endometrial function *in vivo*, and psychological distress might affect the secretion and regulation of the endocrine hormones (18) (**Figure 2**). Furthermore, neurotransmitters and their corresponding receptors are widely distributed across the endometrium (19–22) (**Table 1**). The local content of neurotransmitters in the endometrium and the expression of their receptors might also interfere with the normal function of the endometrium. Therefore, there is a high probability that psychological stress might directly cause endometrial dysfunction. However, the impact of psychological stress on treatment outcomes is unclear, and the specific mechanism of stress-induced fertility disorders is still a mystery. Here, we mainly review the general adaptation strategies and specific mechanisms of endometrial dysfunction caused by psychological stress for female infertility.

**Table 1:** Potential stress mediators in human endometrium.

Stress gene	Location and phase of present	Potential function	References
*BDNF*	Human endometrium	Regulate endometrial cells proliferation	(23)
*NR3C1*	Uterine epithelial cell line	Immune cell trafficking and embryonic development	(24)
*NPY*	Decidua	Immunoreactive of fetal membranes	(25)
*HSPA5*	Human endometrium	Response the paracrine and autocrine factors	(26)
*MAPK*	Human endometrium	Immunomodulatory, regulate stress adaptation and endometrial remodeling	(27)
*PAR2*	Human endometrium	Endometrial remodeling	(28)
*NET*	Late proliferative epithelium	Decidualization	(23, 29)
*EMT*	Secretory stroma	Decidualization	(23, 29)
*VMAT2*	Proliferative stroma and secretory epithelium	Proliferation and decidualization	(23, 29, 30, 31)
*PMAT*	Proliferative stroma	Stromal proliferation	(23, 29, 30, 31)
*PNMT*	Human endometrium	Uterine activity during pregnancy and parturition	(22, 32)
*COMT*	Human endometrial stroma	Inhibit endometrial stroma cell proliferation	(22, 33)
*MAO-A*	Human endometrium	Embryo implantation and endometrial receptivity	(34, 35)
*MAO-B*	Human endometrium	Endometrial receptivity	(34)
*ADRA2C*	Human endometrium	Unclear	(36)
*Opioid receptor*	Human endometrium	Proliferation and decidualization	(37, 38)

## The Role Of Human Endometrium In Reproductive Health

The endometrium is a complex multicellular tissue that dynamically remodels to create a microenvironment suitable for supporting pregnancy (39, 40). The embryo implantation occurs in the functional layer of the endometrium, which is regulated by changes in the ovarian hormones, mainly progesterone and estrogen. In a healthy endometrium, progesterone and estrogen signals are tightly coordinated to drive a normal menstrual cycle and enhance the receptive status of the endometrium for embryo implantation in the implantation window. The local levels of autocrine and paracrine molecules change during the menstrual cycle and play an important role for directing the establishment of uterine receptivity (41, 42). During the process of blastocyst implantation into the uterus, the endometrium stromal bed implanted by the blastocyst undergoes extensive differentiation, revascularization, and recruitment of immune cells. This process is called “decidualization”, which is essential for a successful pregnancy (8). In humans, the rate of natural conception per cycle is poor (about 30%), and 75% of failed pregnancies are due to implantation disorders (43). Previous studies have shown that impaired endometrial stromal proliferation and differentiation are associated with recurrent miscarriage, preeclampsia, intrauterine growth restriction, and unexplained infertility in the clinical setting (44, 45).

### The Dynamic Changes in Human Endometrium

**Figure 1:**
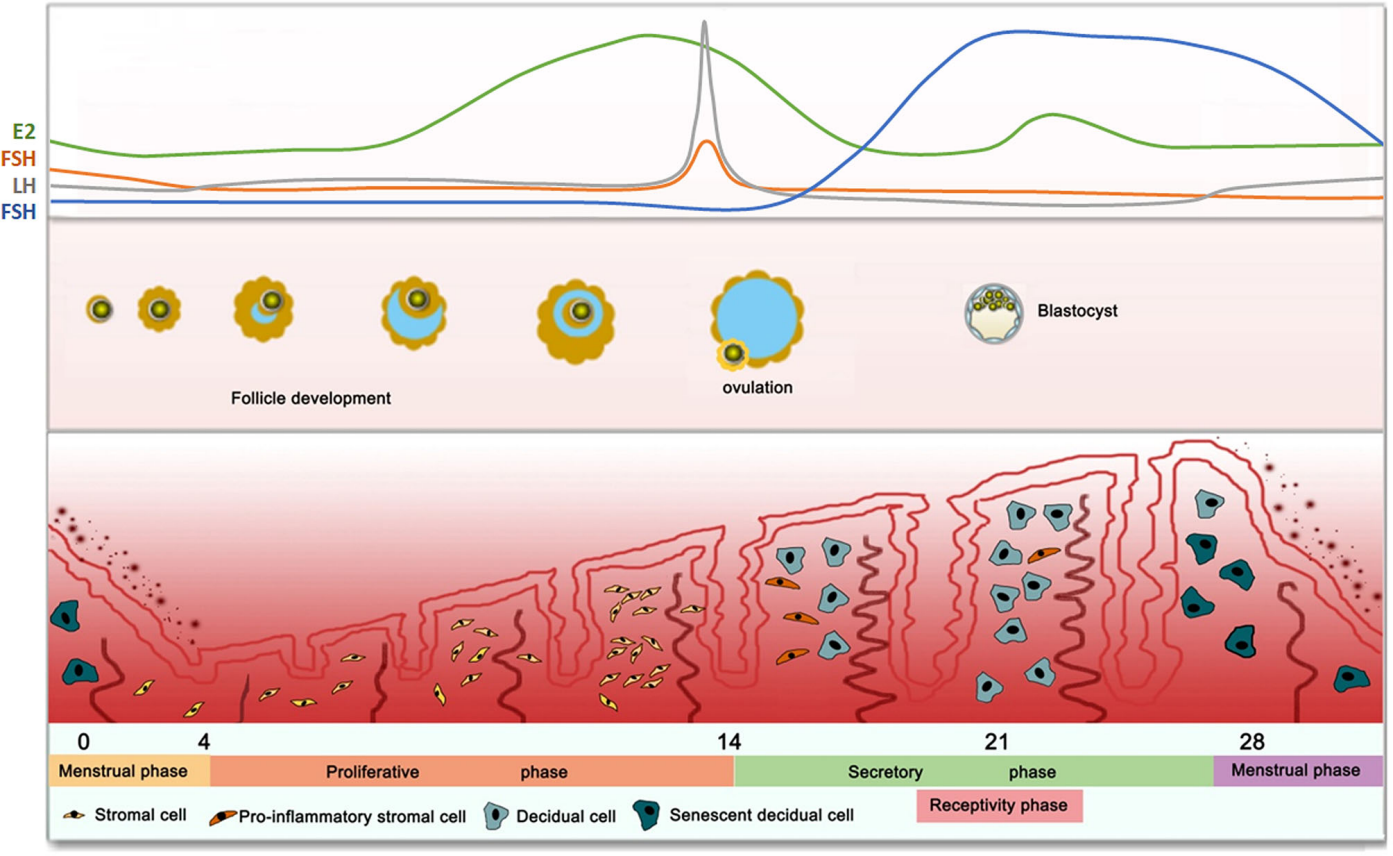
Dynamic Changes of Human Endometrium in Response to Hormones.

In humans, female reproductive physiology involves a complex interaction between neuroendocrine and endocrine signals that are affected by the hypothalamus, pituitary gland, and ovaries. The hypothalamic-pituitary-ovarian (HPO) axis is a tightly regulated system that controls the human endometrium (**Figure 1**). The hypothalamus secretes gonadotropin-releasing hormone (GnRH) pulses, which activate the pituitary to release two gonadotropins, namely follicle-stimulating hormone (FSH) and luteinizing hormone (LH) (46, 47). FSH and LH in turn, act on the ovaries to stimulate follicle growth and produce high level of progesterone after ovulation (48). During the proliferative phase (follicle phase), an increase in the pituitary gonadotropin levels, results in the development of follicles as well as the selection of dominant follicles. The developing ovarian follicles produce estrogen, which promotes the proliferation of epithelial cells, interstitium and vascular endothelium, thereby regenerating and thickening of the endometrium. In the middle of the menstrual cycle, there occurs a surge in LH gonadotropins, resulting in ovulation. Then, the endometrium enters the secretory phase under the action of the continuously increasing progesterone 4 (P4). The early secretory (luteal) phase is characterized by the rupture of the follicle to form the corpus luteum (CL), followed by the secretion of P4 to prepare for implantation (49). During this period, the endometrial stromal cells undergo differentiation (pre-decidualization) to prepare for embryo implantation (50). In the middle secretory (luteal) phase (cycle days 20–24), the elevated estrogen (E2) levels along with P4 define the window of receptivity, which implies uterine receptivity conducive to implantation and subsequent pregnancy (44). Endometrial stromal fibroblasts are transformed into specialized secretory decidual cells, which provide essential nutrients and immune-privileged substrates for embryo implantation and placental development. In the late secretion phase, if there are no surviving embryos, the superficial layer becomes denser, and leukocyte infiltration begins 2-3 days before menstrual shedding, thus resetting the cycle (cycle 0/28 days). On the contrary, implanted blastocysts secrete lutealtrophic factors such as chorionic gonadotropin to sustain the progesterone production in, thereby supporting pregnancy.

### The Role of Endometrial Receptivity in Embryo Implantation

Pregnancy is a physiological process involving multiple independent and cascading performance events, such as embryo implantation, decidualization, placental development, and final fetal delivery (43). The successful completion of the previous event is a prerequisite for the occurrence of the latter event. The progesterone and estrogen secreted by the ovaries are known to precisely control the orderly occurrence of these events. Here, we emphasize the research progress of implantation from the perspective of the endometrium, which is usually a barrier to implantation.

For more than 80 years, scientists have been interested in investigating endometrial receptivity, leading to an in-depth understanding of embryo-endometrial cross-talk and implantation-related processes. In humans, the endometrium receptivity is optimum during the mid-luteal phase of a regular cycle, thus, the LH surge before ovulation occurs between days 6-10 (51, 52). The endometrium is composed of two major tissue compartments: epithelial cells and stromal cells. During the receptivity establishment stage, the epithelial and stromal cells undergo proliferation and differentiation to adapt to embryo implantation as well as post-implantation embryonic development. The receptivity of the endometrium is a complex process that facilitates the attachment, invasion, and development of the embryo. After ovulation, there occur an increase in the levels of progesterone and local cyclic adenosine monophosphate (cAMP). Endometrial stromal fibroblasts transform into specialized secreting decidual cells. The decidual process passes through different phenotypic changes to support the receptivity of the endometrium, embryo selection, and finally, pregnancy resolution (6). Previous studies have shown that several molecules are expressed and silenced in the uterus during the peri-implantation period, which guides the sequential proliferation and differentiation of different cell types in the uterus (45, 53–55). Further analysis has shown that defects in uterine receptivity, such as failure of epithelial clearance and aberrant expression of uterine receptivity-related genes, are the main causes for implantation defects (56, 57).

### Potential Factors Affecting Endometrial Function

Molecular evidence indicates that locally produced signaling molecules, including cytokines, growth factors, homeobox transcription factors, lipid mediators, morphogens and ovarian hormones, act as autocrine, paracrine, and juxtacrine factors to determine the uterine receptivity (58). In this section, we discuss a novel signaling network involving cytokines, homeotic proteins, and morphogens upon implantation. The principal hormones that guide uterine receptivity are ovarian P4 and E2. P4 is produced by the CL in the ovary, and it facilitates embryo implantation and maintenance of pregnancy. In the peri-implantation period before embryo attachment, P4 signaling mediates uterine epithelial growth arrest and stroma proliferation, which is called uterine proliferation-differentiation transition (PDS). Uterine PDS is an important hallmark of uterine receptivity. Some genes, such as HAND2 and BMI1, participate in uterine PDS by modulating P4-PR signaling (59). In mice, estrogen and progesterone act through the estrogen receptor (ER) and progesterone receptor (PR) expressed in the nucleus. Both receptors have two different subtypes, namely ERα/ERβ and PRA/PRB (60). Both subtypes of PRs are expressed in the uterus (61). Gene knockout of PRA and PRB in female mice results in infertility, mainly due to multiple functional defects of the ovary and uterus, however, these functions are normal in female mice lacking PRB alone, indicating that progesterone-mediated uterine function is mainly mediated by PRA (62–64). ERα and ERβ are also expressed in the uterus of mice (61), ERα–/– uteri are hypoplastic and unable to support implantation, whereas ERβ–/– uteri retain biological functions that allow normal implantation (65, 66). This suggests that ERα plays a dominant role in the uterus. Although gene knockout of ERβ does not cause reproductive defects, studies have shown that they may still be involved in the pregnancy process. For example, the expression of ERβ in the endometrium suggests that it might regulate changes in angiogenesis and vasodilation (67, 68). Correspondingly, women with infertility exhibit a significantly reduced expression of ERβ (69, 70). In addition to nuclear receptor transcription factors, such as ER and PR, studies on knockout mice have shown that multiple transcription factors, including Hox family transcription factors, Msx transcription factors, and Kruppel-like transcription factors, also regulate uterine receptivity (71–78). In the reproductive system, Hox genes are known to be widely involved in the development of mouse and human reproductive systems, and consequently in the maintenance of early pregnancy.

Many important signaling pathways are known to regulate cell proliferation, differentiation, and apoptosis, as well as endometrial function. Different from most mammals, before the establishment of endometrial receptivity, human endometrial stromal cells (HESCs) initiate differentiation (pre-decidualization) stimulated by progesterone in the absence of embryos (6, 44, 50). The increased levels of progesterone and local cAMP promote the process of transformation of HESCs into specialized secretory decidual cells, which is a key step in the establishment of endometrial receptivity. The activity of adenylyl cyclase in the endometrium is higher than in the myometrium, CL, or fallopian tube (79). Unlike other types of cells, the continuous increase in intracellular cAMP concentration is critical to the decidualized status in HESCs (80). PKA signaling establishes a feed-forward mechanism, including the selective down-regulation of regulatory PKA subunits during the decidualization of HESCs (81). Other major pathways and kinases activated during the differentiation of HESCs include the WNT/-catenin and JAK-STAT pathways (82–84), Notch signaling pathway (85), ERK1/2 pathway (86, 87), AKT pathway (also known as protein kinase B) (88) and c-Src pathway (89). Meanwhile, stress-induced signaling pathways, such as the JNK and p38 pathways, are completely inhibited when HESCs differentiate into decidual cells (90). Experimental evidence from knockout mice has suggested that Wnt4 might be involved in the proliferation of uterine stromal cells (91). Bone morphogenic protein (BMP), a member of TGF-β signaling, functions through related receptors expressed on the cell membrane. The TGF signaling pathway is known to be involved in regulating the differentiation of uterine epithelial cells (92). ALK3, a member of the BMP protein receptor family, the uterine conditional knocked out ALK3, leading to the continuous proliferation of the uterine epithelium. During the establishment of the endometrial receptive state, the microvillus did not disappear, leading to the abnormal establishment of the receptive state and the failure of implantation (92, 93).

## The Speculative Mechanism Of Stress-Induced Endometrial Dysfunction

The previous section describes that the HPO axis-mediated secretion of hormones determines the functional differentiation and response of the endometrium. Also, various paracrine and autocrine regulatory molecules combined endocrine hormones drive the functional differentiation of the endometrium during the menstrual cycle. Any factors that lead to changes of paracrine and autocrine that regulate endometrial function will cause the dysfunction of the endometrium. In recent years, studies have suggested the role of psychological stress in triggering unexplained infertility and miscarriages may be associated with dysfunction of the hypothalamic-pituitary-adrenal (HPA) axis. The abnormal abundance of neurotrophic factors, sex steroids, metabolic and/or inflammatory cytokines can cause alterations in neurotransmitters, intracellular signaling, gene transcription, and translation. Epigenetic change is a regular and natural occurrence in the regulation of gene expression and activity. It has been proposed that epigenetic changes also contribute to the short-term and long-term imbalances in neuronal function and behavior (15, 94), which may interfere with the normal functioning of the endometrium. The World Health Organization recognizes that women and men suffer considerable psychological pain when they encounter reproductive health problems, including low self-esteem, isolation, loss of control, lack of sexual performance, and depression. The psychological symptoms associated with infertility are consistent with those associated with other diseases, such as cancer, cardiac rehabilitation, and high blood pressure (95). Studies performing the stress in pregnant mice have enabled the exploration of the potential mechanism of stress-induced abnormal pregnancy in mice. Next, we review the role of hormones and local micro-environmental changes in stress-related endometrial dysfunction.

### Psychological Stress Interferes With the Secretion of Endocrine Hormones

**Figure 2:**
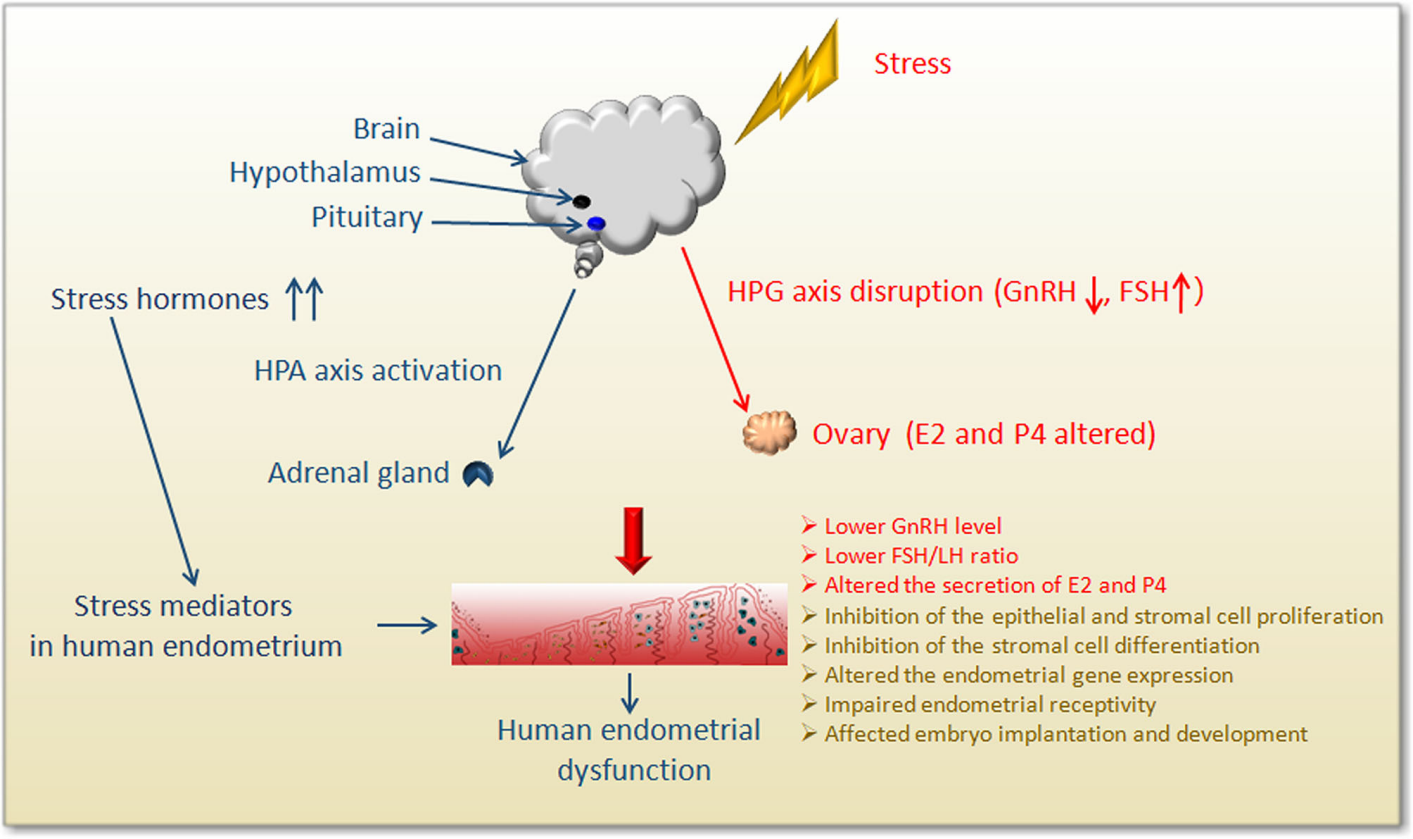
Schematic diagram showing the impact of stress on human endometrium.

The primary endocrine components involved in the response of the stress system include the HPA axis and locus coeruleus-norepinephrine (LC/NE) autonomic nervous system (96) (**Figure 2**). Within a few minutes of stress-induced activation of the HPA axis, the medial parvocellular region of the paraventricular nucleus of the hypothalamus stimulates the release of corticotropin-releasing hormone (CRH) from axonal terminal boutons in the median eminence, which in turn stimulates the corticotrophs in the anterior pituitary gland to release adrenocorticotropic hormone (ACTH) into the systemic circulation (97, 98). When the brain detects a homeostasis challenge, it activates the sympathetic nervous system (SNS), releasing catecholamine-epinephrine (EPI) and norepinephrine (NE), resulting in physiological stress response. This is followed by slow activation of the HPA axis.

Stressors affect all levels of the hypothalamic-pituitary-gonad axis (HPG) (**Figure 2**), resulting in a decrease in the frequency and amplitude of GnRH and LH pulses and a delay in the LH mid-luteal surge, which interferes with the temporal process of reproductive hormone release during the follicular period. These results suggest that the stressors modulate the hypothalamus or the higher centers of the brain (99). Stress can interfere with reproductive function at all levels of the reproductive axis, inhibit sexual desire, reward, and mating behavior at the brain level, especially in the ventral tegmental area, interfering with the release of GnRH pulse generator in the hypothalamus as well as LH and FSH release from the anterior pituitary (100). Different stressors are known to activate different pathways at different duration to affect our health, and the stress adaptation response is affected by the dominant role of certain sex steroids in the circulation (101). Studies have revealed that acute stress can inhibit the HPO axis by inhibiting the secretion of GnRH, thereby inhibiting the release of pituitary LH in female mice (102). Acute injections of CRH into the lateral ventricle of female rats without gonads and adrenal glands showed a rapid and sustained dose-related LH suppression, without the inhibition of FSH secretion (103). Additionally, daily injections of CRH in female rats for the first 12 days after mating was shown to cause 40% of pregnancy interruptions, indicating that CRH directly affected reproductive function in the absence of circulating steroid hormones derived from the adrenal gland and/or gonads (103). A study analyzed the effects of CRH on estradiol and progesterone released from granulosa cells obtained from women undergoing IVF, and found that CRH could significantly reduce media E2 and P4 levels (104). Another study demonstrated CRH significantly inhibited the production of estradiol (E2) and progesterone (P4) in rat and human granulosa cells *in vitro*, acting through the CRH receptor (105). In acutely stressed rats, alteration of sexual behavior, a significant increase in plasma ACTH, prolactin (PRL), corticosterone, and progesterone, and a decrease in FSH were observed (97). The response to acute stress is known to be a highly adaptive phenomenon that enables the individual to properly combat the stressor and recover. However, if the stressor becomes chronic, it can result in complications (106–108). Chronically elevated glucocorticoids (GCs) can result in various complications, including obesity, memory impairment, and mood disorders, as well as affects fertility (100). Chronic stress prolongs this adaptive shift towards a generalized catabolic state. Therefore, sustained HPA hyperactivity gradually leads to a decrease in lean body mass, an increase in visceral fat, and insulin resistance. However, the specific mechanisms of stress-induced endocrine changes and abnormal reproductive function are still worthy of in-depth study and discussion.

### Psychological Stress Acts on the Local Endometrial Microenvironment

The regulation and integration of biological, psychological, and social factors in human physiology benefit from the analysis of precise biomarkers that indicate accurate stress responses. In the past decade, there has been an extensive growth in the use of RNAi in cells, as well as transgenic, knockout, and conditional knockout mouse models, which provide valuable insights into the stress-induced endometrial dysfunction. Additionally, psychological stress not only interferes with the functioning of the endometrium by affecting endocrine hormones but also directly regulates the local microenvironment of the endometrium.

#### Estrogen and Progesterone-Related Stress Molecules in the Endometrium

The human endometrium contains specific cells that undergo cyclic changes under the influence of steroid hormones as well as numerous local paracrine and autocrine factors. The stressors trigger the endometrial endoplasmic reticulum (ER) stress to deplete energy and cause periodic changes in the menstrual cycle (109). Emerging evidence has confirmed that heat shock 70 KDa protein (HSPA5, also known as GRP78/BiP), a molecular chaperone within the ER, plays a crucial role in stress conditions, and is dynamically expressed throughout the menstrual cycle, and has been found to be closely related to the levels of E2 in the normal human endometrium (26). The mitogen-activated protein kinase (MAPK) cascade mediates cellular responses to environmental signals and helps cells adapt to high temperatures, which is a prerequisite for mammalian pathogens (110). During the secretory phase of the menstrual cycle, prolactin (PRL) and PRL receptors are expressed in human endometrial tissue, which, in turn, stimulates the tyrosine phosphorylation of the human endometrial MAPK/ERK pathway (27). Protease-activated receptor 2 (PAR2) is also expressed in the endometrium, which can activate downstream MAPKs and plays an important role in human endometrial remodeling (28). Unequivocal evidence indicates that in human endometrium stromal cells, the nuclear PGR mediates rapid nongenomic progestin responses, which in turn, triggers rapid MAPK and AKT activation in response to progestin signaling (111).

#### Human Endometrial Adrenaline-Related Molecules

The expression of the mouse uterine adrenergic receptor Adrb2 temporarily increases during the peri-implantation period. Abnormal activation of the receptor Adrb2 signal can destroy the space between embryos during implantation. The activation of the cAMP-PKA pathway can lead to a significant increase in the loss of mid-pregnancy and is known to be accompanied by a specific down regulation of LPA3 (19). Previously, the LPA3 gene was known to be essential for uterine contraction and embryo spacing (112). Abnormal Adrb2 activation in early pregnancy provided a molecular clue that explained how early maternal stress could adversely affect pregnancy outcomes (113). Adrenergic receptors play an important role in promoting angiogenesis and maintaining normal embryonic development at the maternal-fetal interface. A study found that the selective knockout of the adrenergic receptors Adra2a, Adra2b, and Adra2c in the mouse placenta blocked the activation of downstream ERK signaling, leading to defects in the development of blood vessels in the yolk sac and placental labyrinth layer, in turn affecting the exchange of nutrients and gas between the mother and fetus, leading to death between E9.5 and E11.5 (114). Human endometrial and ectopic endometrial tissues exhibit a sympathetic nerve distribution similar to the rat uterus (115). A study used the single-cell transcriptome sequencing technology to analyze the difference in gene expression of human endometrial stromal cells before and after differentiation and found that the expression of ADRA2C and monoamine-related transporters in decidualized stromal cells presented a dynamic expression pattern, which gradually increased with the process of decidual differentiation (36). This result indicated that the human endometrium could regulate and accept adrenergic nerve signals. Various interactions between the signaling pathways of the stress and depression systems, especially the growth factor signaling pathway and Akt, S6K, GSK3β, and mTORC1 signaling (94, 116) have been confirmed to be involved in the decidual transformation process of endometrial stromal cells (117–121).

#### Stress-Related Neurogenic Molecules Participate in the Regulation of Endometrial Function

During the process of embryo implantation and pregnancy preparation, uterine epithelial cells undergo genomic and biological transformations, which mediate the adhesion and invasion of blastocysts. GCs is an important mediator involved in psychological stress. The whole-genome microarray analysis of a human endometrial cancer cell line revealed that GC and GC receptor were expressed in the uterine epithelial-like cell line (NR3C1). This suggested that GC signaling regulated important biological functions, including immune cell trafficking and embryonic development (24). Brain-derived neurotrophic factor (BDNF) is a stress-related gene and a member of the neurotrophic growth factor family (122). Recent studies have shown that BDNF levels play an important role in regulating the proliferation of endometrial cells (123). Endogenous opioids play an important role in regulating stress-related behaviors. Research has defined the dynamics of the expression and localization of the opioid receptor in human endometrium throughout the menstrual cycle (37). In addition, Kappa opioids exerted a time- and dose-dependent inhibitory effect on TGF-β1 production from endometrial stromal and epithelial cells (38). Neuropeptide Y (NPY), a neuroendocrine/peptide mediator that coexists with NE in many sympathetic nerves and participates in the regulation of psychological stress was found in maternal decidua and fetal membranes (25). Additionally, recent studies showed that high-dose NPY could inhibit the proliferation of human adipose-derived stem cells and promote their differentiation (124). Researchers expected used NPY to repair the human endometrium and treat endometrial damage-induced female infertility (125). The uterus is innervated by adrenergic sympathetic nerve fibers, and the endometrium can synthesize endogenous monoamines. Several studies have shown that monoaminergic neurotransmission involving serotonin (5-HT), NE, and dopamine (DA) significantly impact the brain circuits related to mood regulation and psychological stress response (126–128). Monoamines play an important role in reproductive processes, such as decidua, implantation, immune regulation, and inflammation, and are an effective vasoactive mediator, regulating blood flow, and capillary permeability (23, 29). Studies on the effects of monoamines have shown that the normal endometrium contains monoamine transporter (EMT), NE transporter (NET), and vesicles throughout the menstrual cycle and early decidua. The vesicle monoamine transporter (VMAT) and plasma membrane monoamine transporter (PMAT) might affect endometrial decidualization and blastocyst implantation by uptake of extracellular histamine and could be subsequently released on demand (23, 29–31). Tyrosine hydroxylase (TH) and phenylethanolamine-N-methyltransferase (PNMT) are vital for NE to synthesize EPI, while catechol-O-methyltransferase Enzyme (COMT) and monoamine oxidase (MAO) are used for metabolizing both NE and enzymes for adrenaline synthesis. They are considered as sympathetic markers, they regulate psychological stress and provide an important physiological mechanism for controlling endometrial activities during pregnancy and parturition (22, 32, 33). MAO is an important psychological stress regulator and is involved in the process of adrenaline metabolism. MAO is widely distributed in the body, exists on the outer membrane of mitochondria, and participates in the pathophysiological regulation of neuropsychiatry-related diseases, including major depression, addiction, and violent psychological diseases. Studies have shown that MAO is highly expressed in the receptive state of the endometrium. The activity of MAO increases significantly in the endometrium and changes periodically during the menstrual cycle (34). The expression and localization of MAO in the endometrium are known to show periodic changes with the changes in the levels of estrogen and progesterone *in vivo* (129). This hormone-dependent temporal and spatial specific expression and localization pattern has been shown to be an important factor in establishing endometrial receptivity. MAO is regarded as an important marker for the establishment of endometrial receptivity, which helps in embryo transfer in clinician-assisted reproduction practice and improves embryo implantation rate (130). The expression of MAO-A in the glands and stromal cells of the human endometrium during the receptive phase has been found to be significantly higher than that in the pre-receptive phase. The expression is reduced during the window in patients with repeated implantation failure, indicating that MAO-A has physiological significance in the preparation of the endometrium for implantation (35). Additionally, MAO inhibitors are considered to be potential contraceptives in rats, which can significantly reduce the pregnancy rate in mice (131).

Thus, psychological stress is closely related to the functioning of the human endometrium. The stressors not only interfere with the reproductive hormones that regulate the endometrium but also directly participate in the regulation of the microenvironment of the endometrium (**Table 1**).

## Conclusions And Future Perspectives

Psychological stress includes the physiological reaction to threat or pressure, which is common in various physical illnesses and is increasingly recognized as a risk factor for disease onset and progression. If left untreated, chronic stress or burnout might develop, resulting in the need for medical assistance. Many studies have indicated that psychological stress might be an important risk factor underlying infertility. Here, we showed that abnormal endometrial function affects the establishment of receptivity and decidual development, leading to female infertility. The current clinical treatments for monitoring pathological endometrium changes include hysteroscopy, transcriptome sequencing of endometrial biopsy to select the best period for embryo transfer, intrauterine perfusion of human chorionic gonadotropin (HCG), uterine scratching before embryo transfer, and stem cell therapy (132–135). However, these invasive treatments have no significant effect on restoring endometrial function and increasing the pregnancy rate. In this review, we systematically analyzed the direct and indirect potential molecular basis of endometrial dysfunction caused by psychological stress in term of endometrial system and local microenvironment. We focused on the impact of psychological stress factors on the female endometrial function which should not be ignored for the treatment of RIF. Studying the interplay between psychological stress and the human endometrium, especially understanding how psychological stress changes the molecular mechanisms and signaling pathways in reproductive system tissues, would provide insight into the specific mechanisms of stress-mediated dysfunction of the human endometrium.

Here, we evaluated the effects of psychological stress on the human endometrium and subsequently on pregnancy outcome. It is necessary to understand how these mechanisms work during gestational stress *in vivo* and to understand how these mechanisms work in the human endometrium. Psychological interventions for women with infertility have the potential to decrease anxiety and depression and may well lead to significantly higher pregnancy rates. Therefore, infertility patients must be psychologically counseled and supported as they go through assisted reproductive technology (ART) treatment. We recommend that the Society of Reproductive Medicine or the Center of Reproductive Medicine to incorporate psychological assessment and intervention into the routine diagnosis and treatment of ART. In addition, how do epinephrine or glucocorticoids caused by stress affect the nervous system and reproductive system? How do the signals between the nervous system and the reproductive system influence and feed back to each other? Is the abnormal reproductive function caused by stress reversible? What kind of psychological intervention is the most effective for infertile couples? These are all worth exploring and answering in the future.

## Author Contributions

J-xW and S-bK prepared the manuscript. SL give an intensive suggestion and discussion. All authors contributed to the article and approved the submitted version.

## Funding

This work was supported by National Natural Science Foundation of China (81901481 to J-xW).

## Conflict of Interest

The authors declare that the research was conducted in the absence of any commercial or financial relationships that could be construed as a potential conflict of interest.

## Publisher’s Note

All claims expressed in this article are solely those of the authors and do not necessarily represent those of their affiliated organizations, or those of the publisher, the editors and the reviewers. Any product that may be evaluated in this article, or claim that may be made by its manufacturer, is not guaranteed or endorsed by the publisher.
